# Electrically-Driven Soft Fluidic Actuators Combining Stretchable Pumps With Thin McKibben Muscles

**DOI:** 10.3389/frobt.2019.00146

**Published:** 2020-01-10

**Authors:** Vito Cacucciolo, Hiroyuki Nabae, Koichi Suzumori, Herbert Shea

**Affiliations:** ^1^Soft Transducers Laboratory (LMTS), Institute of Microengineering, School of Engineering, École Polytechnique Fédérale de Lausanne (EPFL), Neuchâtel, Switzerland; ^2^Suzumori-Endo Laboratory, Department of Mechanical Engineering, Tokyo Institute of Technology, Tokyo, Japan

**Keywords:** soft robotics, soft actuators, soft wearables, soft fluidic actuators, wearable robots, stretchable pumps, thin McKibben muscles, artificial muscles

## Abstract

Soft wearable robots could provide support for lower and upper limbs, increase weight lifting ability, decrease energy required for walking and running, and even provide haptic feedback. However, to date most of wearable robots are based on electromagnetic motors or fluidic actuators, the former being rigid and bulky, the latter requiring external pumps or compressors, greatly limiting integration and portability. Here we describe a new class of electrically-driven soft fluidic muscles combining thin, fiber-like McKibben actuators with fully Stretchable Pumps. These pumps rely on ElectroHydroDynamics, a solid-state pumping mechanism that directly accelerates liquid molecules by means of an electric field. Requiring no moving parts, these pumps are silent and can be bent and stretched while operating. Each electrically-driven fluidic muscle consists of one Stretchable Pump and one thin McKibben actuator, resulting in a slender soft device weighing 2 g. We characterized the response of these devices, obtaining a blocked force of 0.84 N and a maximum stroke of 4 mm. Future work will focus on decreasing the response time and increasing the energy efficiency. Modular and straightforward to integrate in textiles, these electrically-driven fluidic muscles will enable soft smart clothing with multi-functional capabilities for human assistance and augmentation.

## Introduction

Soft wearables for human assistance and augmentation can provide muscle support for lower and upper limbs (Galiana et al., 2012; Asbeck et al., 2014; Natali et al., 2019), decrease the metabolic cost of running and walking (Kim et al., 2019), provide haptic feedback (Hinchet et al., 2018; Takahashi et al., 2019). They should be comfortable to wear and discretely integrated into clothing, however most development on wearable robots has been carried out to date using electromagnetic motors. It is becoming clear that intrinsically soft actuators are required for fully-integrated soft wearables. Of the different classes of soft actuators, soft fluidic actuators are the most widely adopted for wearables, due to their robustness, simple fabrication, and high energy density. These actuators generally consist of a chamber inflated with a pressurized fluid. Examples include bellows-shaped pneumatic actuators (“pneu-nets”) (Mosadegh et al., 2014; Yap et al., 2015), fiber-reinforced fluidic actuators (Polygerinos et al., 2014; Cacucciolo et al., 2016), and McKibben actuators (Wehner et al., 2013).

**Figure 1:**
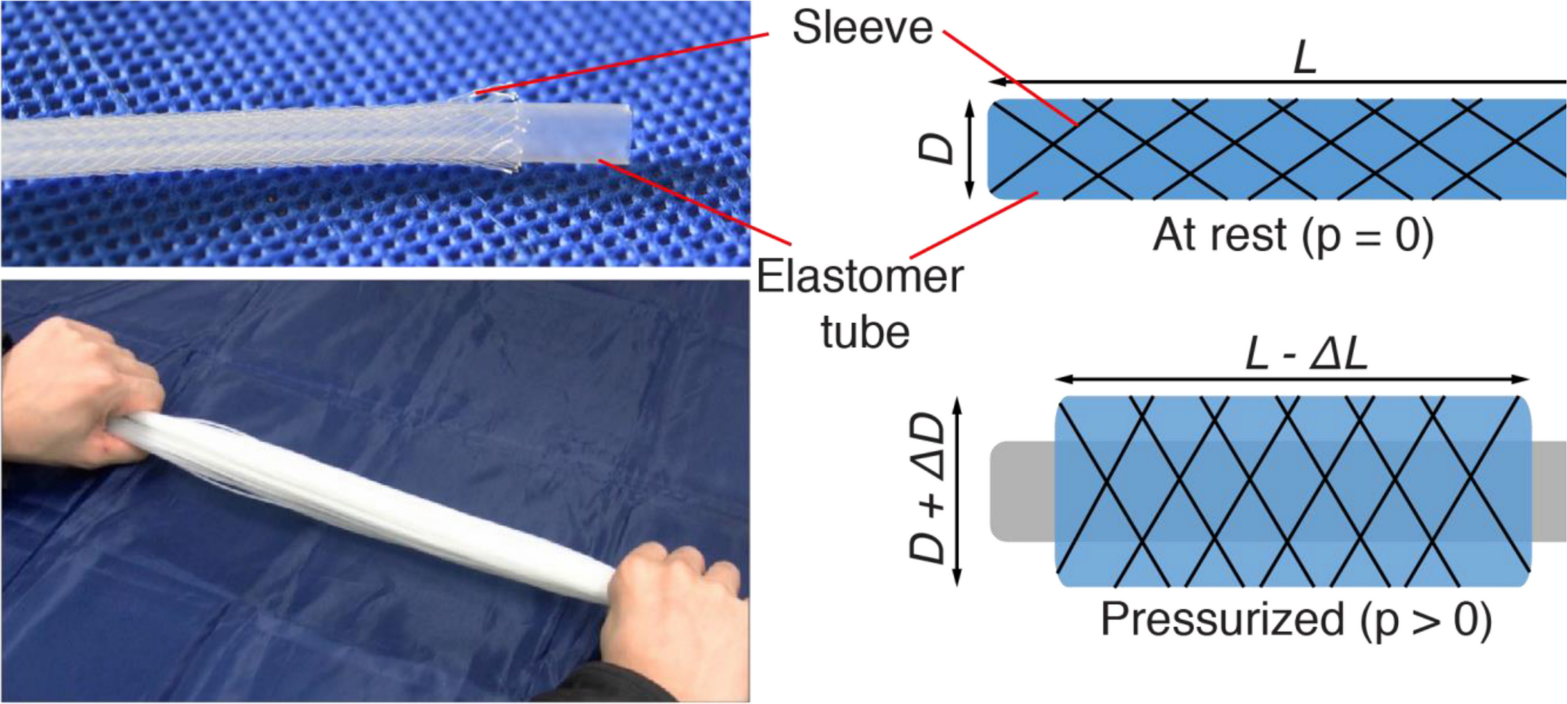
Thin McKibben muscles (Kurumaya et al., 2017). Each filament is composed of an elastomer tube and an external sleeve. When the tube is pressurized, the sleeve drives the deformation into a axial contraction and radial expansion. A number of filaments can be arranged together in bundles to provide high contraction force.

A recent development in McKibben actuators is devices with very small diameter (inner diameter of order 1 mm), called Thin McKibben Muscles (TMMs) for their similarity with mammal's muscles (Kurumaya et al., 2017). TMMs are highly flexible even when pressurized, unlike conventional McKibben actuators, which drastically stiffen with pressurization. TMMs filaments can be bundled together to multiply the contracting force (Figure 1). Such TMMs have been demonstrated in a wide variety of wearable devices for human support and assistance and have been successfully knitted into textiles (Koizumi et al., 2018; Abe et al., 2019).

**Figure 2:**
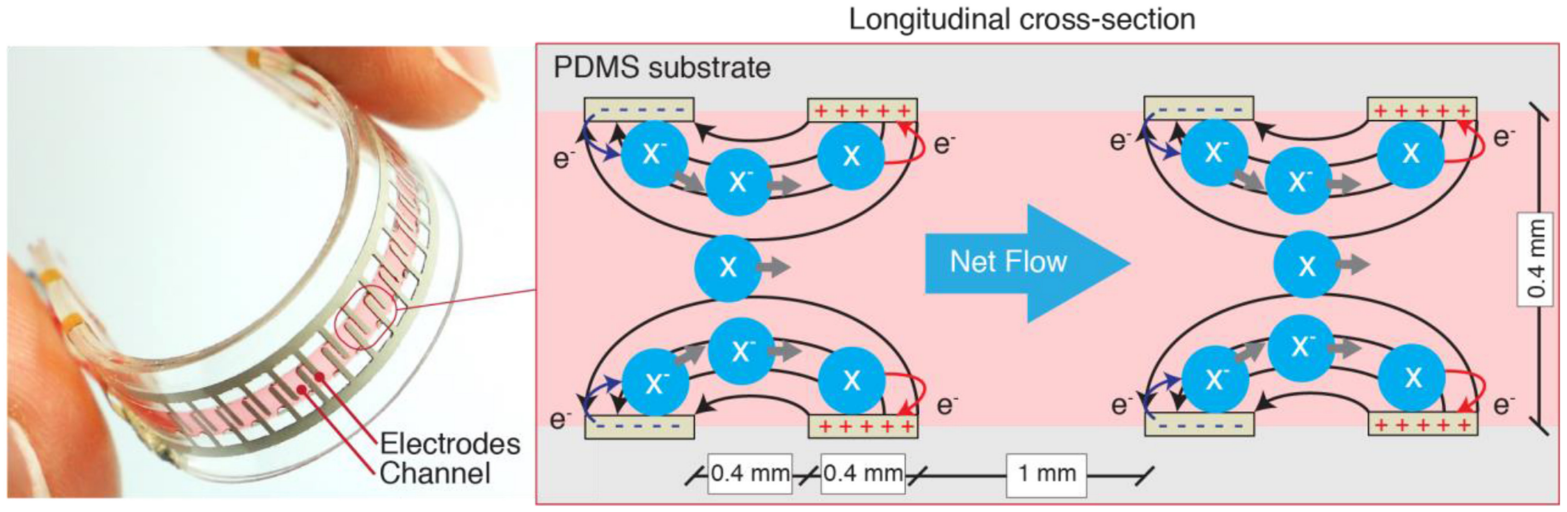
Schematics of the Stretchable Pumps (Cacucciolo et al., 2019) used in this study, based on ElectroHydroDynamics. The electrodes are made of a stretchable silver ink. The top and bottom PDMS substrates are each 400 μm-thick. The pump generates continuous flow with no moving parts.

TMMs have a limitation that is common to all soft fluidic actuators: they require an external pump or compressor to drive the pressurized fluid. Such pumps are generally rigid, noisy and bulky components that cannot be integrated with the actuator, preventing untethered and portable operations. To overcome this limitation, Cacucciolo et al. have recently developed a class of all-soft-matter electrically-driven pumps (Cacucciolo et al., 2019). These 1 g-weighted Stretchable Pumps are solid-state and pump the liquid using a physical mechanism called EHD (ElectroHydroDynamics), where the liquid molecules are directly accelerated by an electric field, without moving mechanical components (Figure 2). These pumps can be largely bent, stretched and twisted while operating, allowing straightforward integration into wearables. The authors demonstrated these pumps in a wearable thermal regulation device and in self-contained bending fluidic actuators.

**Figure 3:**
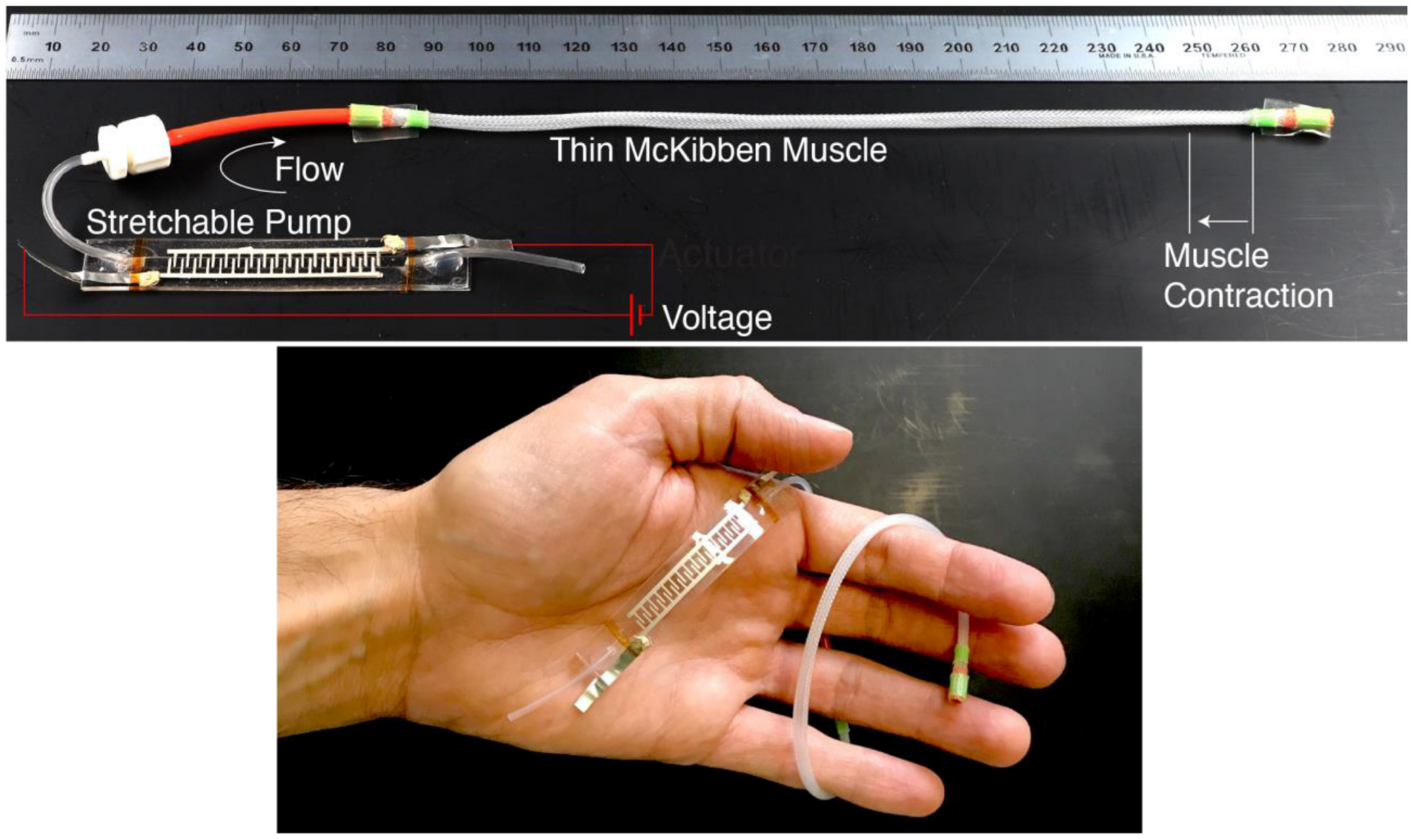
Integration of a Stretchable Pump with a thin McKibben Muscle. When the voltage is turned on, the pump accelerates the fluid by EHD (ElectroHydroDynamics) and drives it into the muscle, causing its contraction. A negative voltage generates opposite flow, with fluid being driven out of the muscle, leading to its relaxation. The result is an electrically-driven soft fluidic muscle.

In this work, we describe the first integration of Stretchable Pumps with thin McKibben Muscles. The two devices together weight 2 g, are slender (maximum radial size 4 mm for the muscle and cross section of 1.5 × 10 mm for the pump), and can be largely bent or twisted (Figure 3). We measured a blocked force of 0.84 N and a maximum contracting stroke of 4 mm (corresponding to 2.2% of the muscle length). The result is an electrically-driven fluidic muscle for integration in soft wearables and active textiles.

This paper is organized as follows. In section Materials and Methods we describe the design and fabrication of the TMM and of the Stretchable Pumps, their integration and the characterization experiments. Section Results reports the characterization of the TMM and of the TMM integrated with the pump, in terms of force vs. contraction ratio (defined as stroke over initial muscle length) and response time. We then present a demo where we repeatedly lift a 2 g weight, equivalent to the weight of the Stretchable Pump—TMM ensemble. In section Discussion we discuss the results, limitations and future developments.

## Materials and Methods

The TMM (Thin McKibben Muscles) used in this study are a modified version of the devices developed by the authors in previous works (Kurumaya et al., 2017; Koizumi et al., 2018; Suzumori et al., 2018; Abe et al., 2019). To enable operation of the TMMs using Stretchable Pumps instead of industrial pressure source required lowering the TMM actuation pressure from 100 to 200 kPa to 10–20 kPa, resulting in LPTMMs (Low Pressure Thin McKibben Muscles). The decrease in actuating pressure was achieved by decreasing the wall thickness of the inner tube. However, it becomes more challenging to obtain tubes of uniform thickness when the thickness values become too low. As a tradeoff, we selected a tube with a wall thickness of 0.15 mm.

The Stretchable Pumps used in this study are based on the design and fabrication of the Ag pumps (i.e., the pump with silver electrodes) presented in Cacucciolo et al. (2019). Figure 2 shows a picture of a Stretchable Pump (left) and the schematic diagram of the working principle (right). The channel and the electrodes backing are made of 400 μm-thick Polydimethylsiloxane (Dow Corning Sylgard 184). The interdigitated electrodes are realized by casting a commercial stretchable silver ink (Chimet Ag 520 EI) through a 23 μm-thick BoPET (biaxially-oriented polyethylene terephthalate) mask. The laser-cut channel layer is bonded to the electrode layers using silicone adhesives.

**Figure 4:**
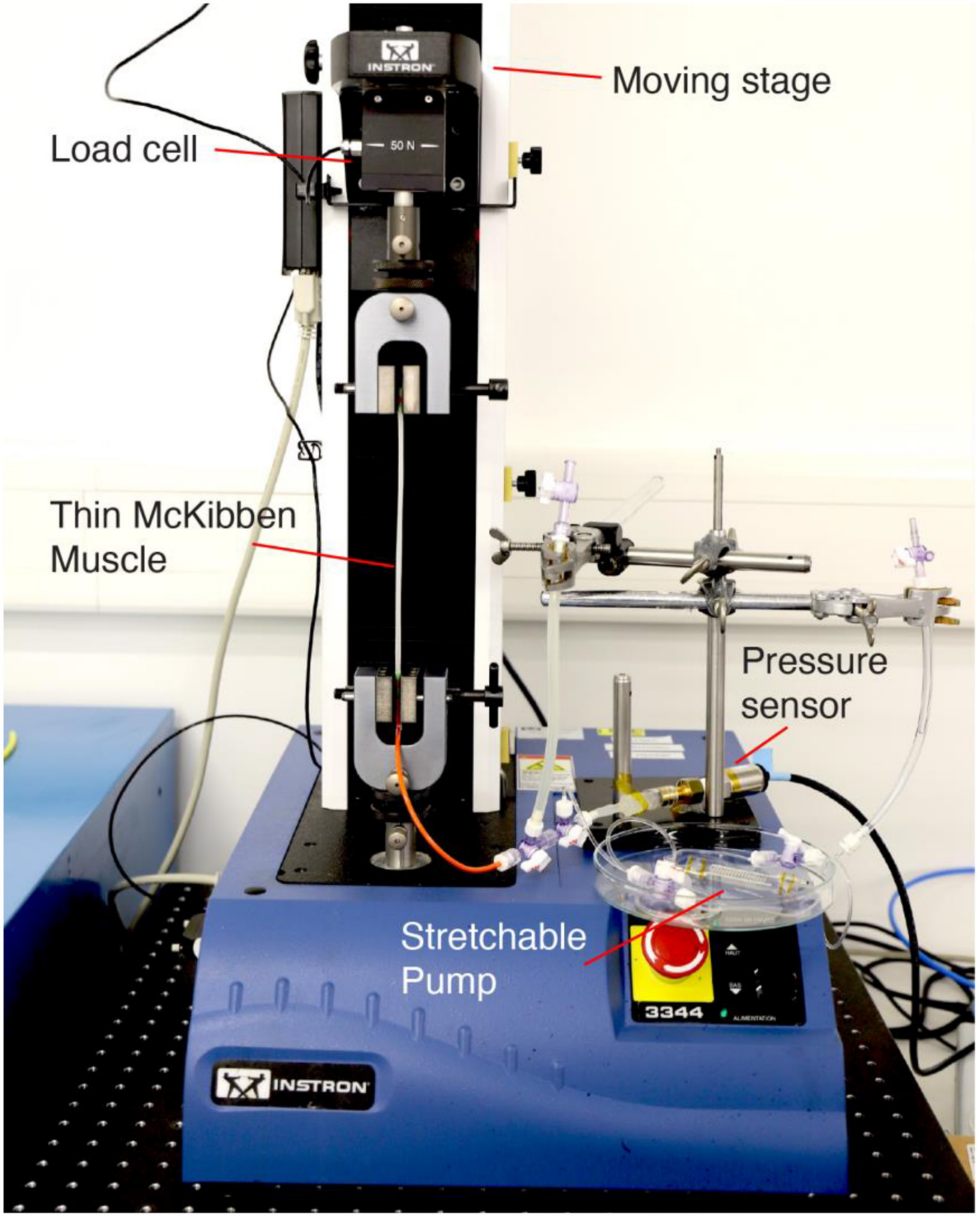
Testing apparatus composed of a pull tester with a load cell and a motorized stage, a pressure sensor, the Stretchable Pump and the LPTMM actuator.

The integration of the Stretchable Pumps with the LPTMMs is achieved by connecting the pump's outlet tube to the actuator's inlet tube through a fluidic connector (Figure 3). We measured the LPTMM force and displacement by using a pull-tester (Instron Universal Testing System). One end of the actuators is mechanically clamped to the fixed side of the pull-tester, while the other end is clamped to the motorized stage through a load cell (Figure 4). The voltage to the Stretchable Pump is provided by an EMCO CB 101 power supply, with voltage and current measurement outputs. An H-Bridge circuit is used to reverse the polarity of the voltage in order to invert the pump flow direction. The pressure output from the pump is recorded using a pressure sensor (SSI Technologies, P51 MediaSensor™).

**Figure 5:**
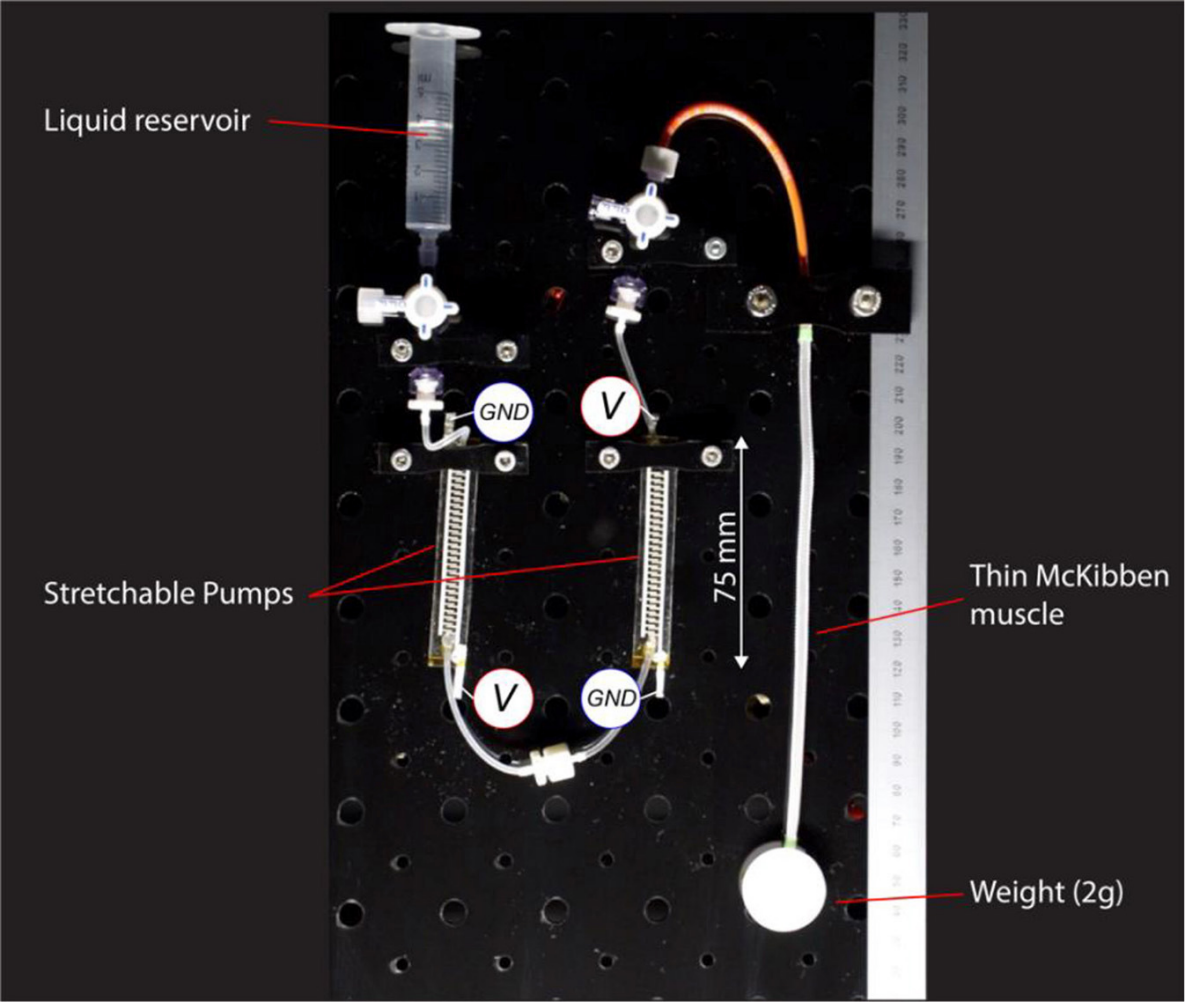
Schematics of the weight-lifting experiments (Supplementary Video 1). Two Stretchable Pumps are connected in series to one LPTMM lifting a 2 g weight.

The load-lifting demo consists of two Stretchable Pumps connected in series between a reservoir and one LPTMM actuator (Figure 5). The free-end of the actuator is connected to a plastic disk weighting two grams. The displacement of the disk is recorded using a digital camera at 50 fps and measured using an image processing software (Kinovea).

## Results

Sections Characterization of Volume vs. Pressure for the LPTMMs and Characterization of Force and Contraction Ratio for the LPTMMs present the characterization of the LPTMMs driven by a pressure generator, while section Characterization of Force and Contraction Ratio for the LPTMMs Driven by Stretchable Pumps. Section Weight-Lifting Demo includes an experiment where one LPTMM driven by two Stretchable Pumps cyclically lift up a weight.

### Characterization of Volume vs. Pressure for the LPTMMs

In sections Characterization of Volume vs. Pressure for the LPTMMs and Characterization of Force and Contraction Ratio for the LPTMMs, we first characterized the LPTMMs using a pressure generator (Fluigent MFCS™) before connecting them to the Stretchable Pumps. The aim of the first tests was to characterize the LPTMMs as fluidic loads, in terms of pressure vs. volume. To subtract the influence of the fluidic circuit (tubes, valves, connectors) and of the eventual air trapped into the system, we first conducted the experiment without the LPTMM. We removed these results from those taken with the LPTMM to obtain the net characteristic of the LPTMM. The results are summarized in Table 1. We measured a pressure—volume characteristics of 0.24 kPa/μl when pressurizing or depressurizing the system. We therefore need 83 μl of liquid to reach a pressure of 20 kPa. This result can be merged with the pressure—flow-rate characteristic of the pump to predict the pump flow-rate required to obtain a given response of the actuator.

**Table 1:** Pressure—Volume characteristic of the thin McKibben muscles and of the fluidic circuit.

	**Pressure—volume slope [kPa/μl]**	
	**Pressurizing**	**Depressurizing**
Fluidic circuit	0.70	−0.65
Fluidic circuit + muscle	0.18	−0.18
Muscle only	0.24	−0.24

### Characterization of Force and Contraction Ratio for the LPTMMs

**Figure 6:**
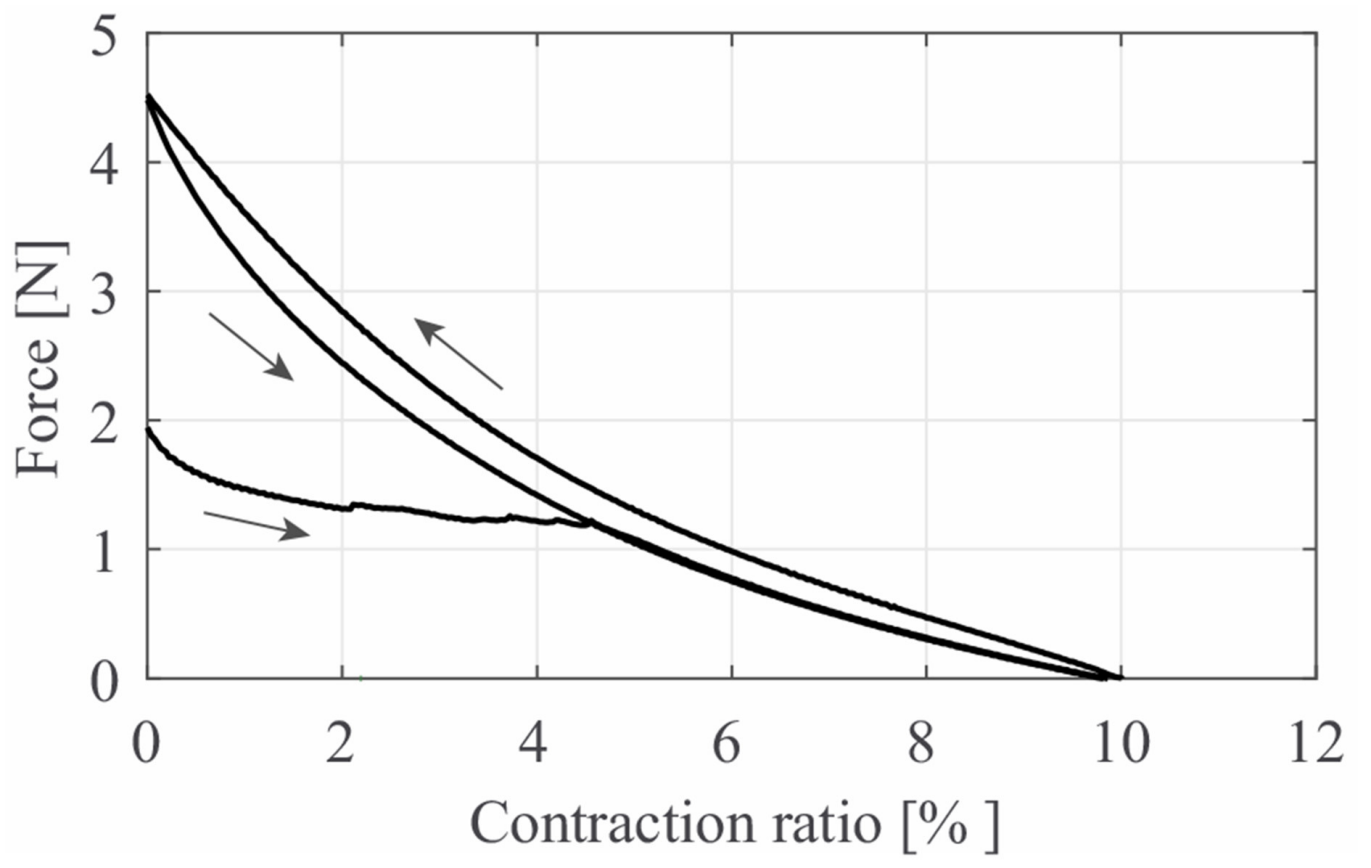
The typical quasi-static characteristic curve of thin McKibben muscles, obtained using a pressure generator. The curve branch on the bottom left applies only to the first actuation cycle after pressurization. After, the actuator consistently follows the cycle on the right.

Figure 6 shows the typical characteristic curve of LPTMMs at 50 kPa. For a given pressure, a LPTMM behaves as a non-linear spring. We performed this measurement by mounting the LPTMM in a pull tester. We apply the pressure with the actuator at its rest length *L*_0_ = 180 mm (length corresponding to ambient pressure) and measure its first contraction force *F*_*FC*_. The pull tester then gradually decreases the length of the actuator until reaching a zero contraction force. The branch on the left of Figure 6. represents the behavior of the actuator during this first phase. The contraction value Δ*L* at zero force represents the free contraction of the actuator at that given pressure. It follows one full cycle of elongation back to the initial length *L*_0_, at maximum force (blocked force *F*_*B*_) and then shrinking back until zero force, at maximum elongation. After the first contraction, the actuator always follows the same cycle, showing very high repeatability (maximum standard deviation of 0.0312 N among five trials). The reasons for the lower force values at the first contraction are related to the sliding between the inner chamber and outer sleeve of the LPTMMs and related friction each time the soft muscles are fully depressurized and pressurized again (Kurumaya et al., 2017; Koizumi et al., 2018; Abe et al., 2019).

**Figure 7:**
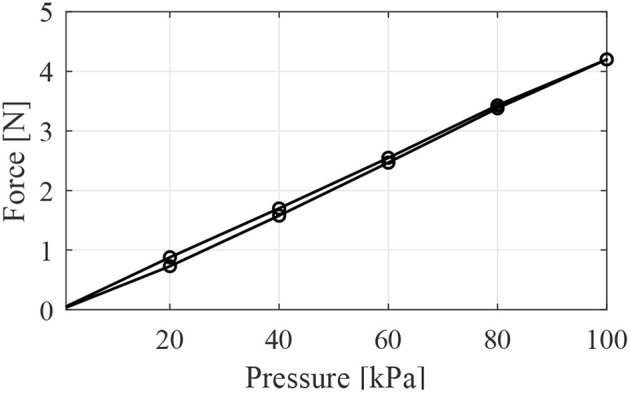
Force vs. pressure response curve of one thin McKibben muscle obtained using a pressure generator.

Figure 7 shows the force vs. pressure results for the LPTMMs. As expected for McKibben muscles, the curve is almost linear and there is very little hysteresis (Kurumaya et al., 2017; Koizumi et al., 2018; Abe et al., 2019). We measured a force vs. pressure characteristic of 0.0424 N/kPa. The measured force in Figure 7. corresponds to the contraction force at the first pressurization. It is lower than the blocked force that the LPTMM can achieve after being contracted and stretched again, as shown in Figure 6.

**Figure 8:**
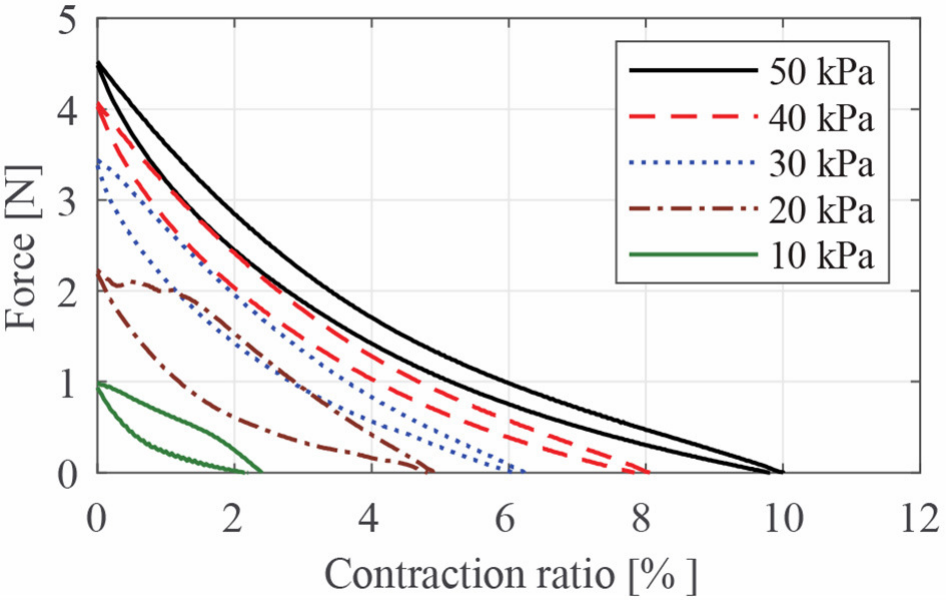
Quasi-static characteristic curves of the thin McKibben muscle used in this study, obtained using a pressure generator.

Figure 8 presents the characteristic curves of LPTMM at different pressures. We removed the left branches corresponding to the first pressurization for clarity. The area below each of these curves represents the energy per unit length of the LPTMM. As expected, higher pressures lead to both higher blocked forces and higher free contraction values, leading to higher energy. We can notice that, unlike the contraction force at initial pressurization (Figure 7), the maximum blocked force shown in Figure 8 increases less than linearly with the pressure, possibly due to the stiffening of the inner tube elastomer and due to friction between the inner tube and the outer sleeve.

### Characterization of Force and Contraction Ratio for the LPTMMs Driven by Stretchable Pumps

We measure force and contraction ratio of a Stretchable Pump connected to a LPTMM using the same set-up described for the characterization of LPTMMs (sections Characterization of Volume vs. Pressure for the LPTMMs and Characterization of Force and Contraction Ratio for the LPTMMs). The Stretchable Pump replaces the pressure generator. The pump is activated using a DC voltage source. When the voltage is turned on, the pump moves the liquid from a reservoir to the LPTMM. The main difference between the pressure generator and the Stretchable Pump is that the former imposes a constant pressure regardless of volume change in the LPTMM, while the pumps pressure depends on flow-rate. As a consequence, the dynamics of the Stretchable Pumps will influence the characteristic curves of the LPTMM.

**Figure 9:**
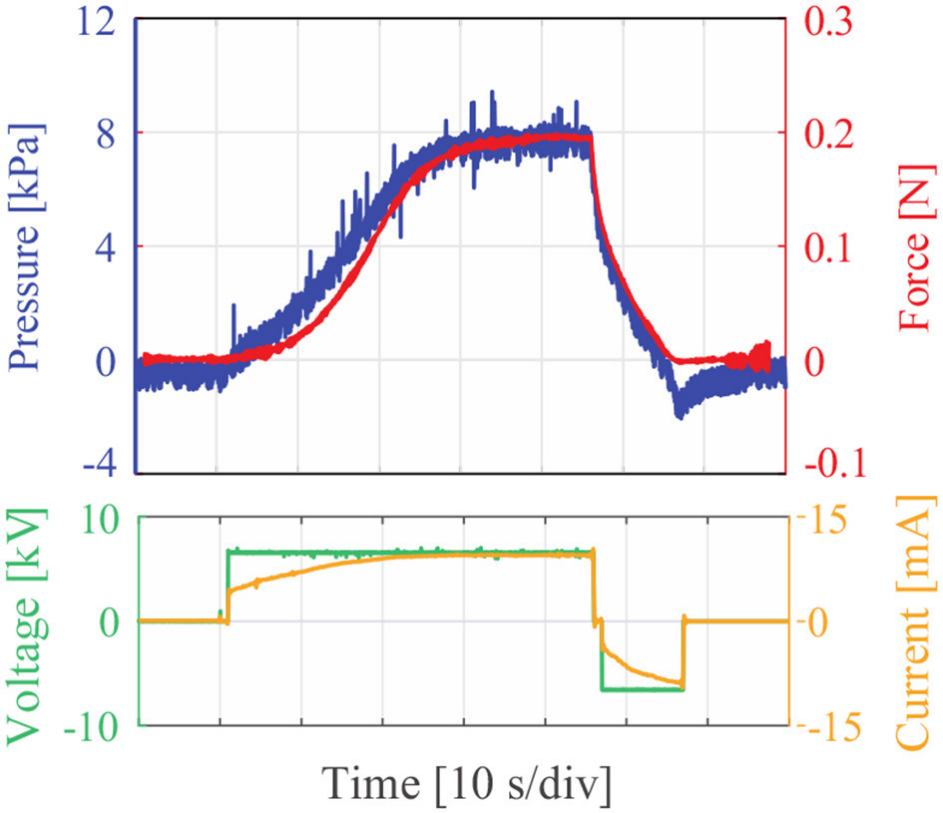
Response curve of one Stretchable Pump and one thin McKibben muscle to a voltage step.

We measure the dynamic response to a 7 kV voltage step of a Stretchable Pump connected to a LPTMM. The pressure response gives a good approximation of the dynamics of the Stretchable Pump. The time required to achieve the maximum pressure here is determined by both: (1) the intrinsic dynamics of the Stretchable Pumps that use FC-40 as dielectric liquid, (2) the fluidic characteristics of the LPTMM (section Characterization of Volume vs. Pressure for the LPTMMs), since at higher flow rate values of the Stretchable Pumps correspond lower pressure values. The generated force replicates closely the pressure profile, consistent with the data presented in Figure 9. The force here is the first contraction force (see section Characterization of Force and Contraction Ratio for the LPTMMs).

**Figure 10:**
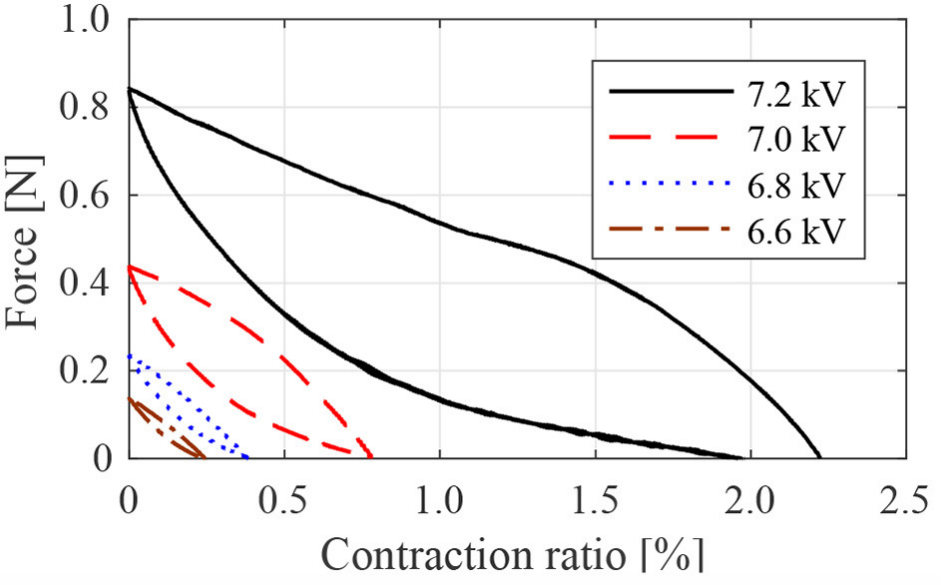
Quasi-static characteristic curves of one thin McKibben muscle activated with one Stretchable Pump, at different values of the applied voltage.

**Figure 11:**
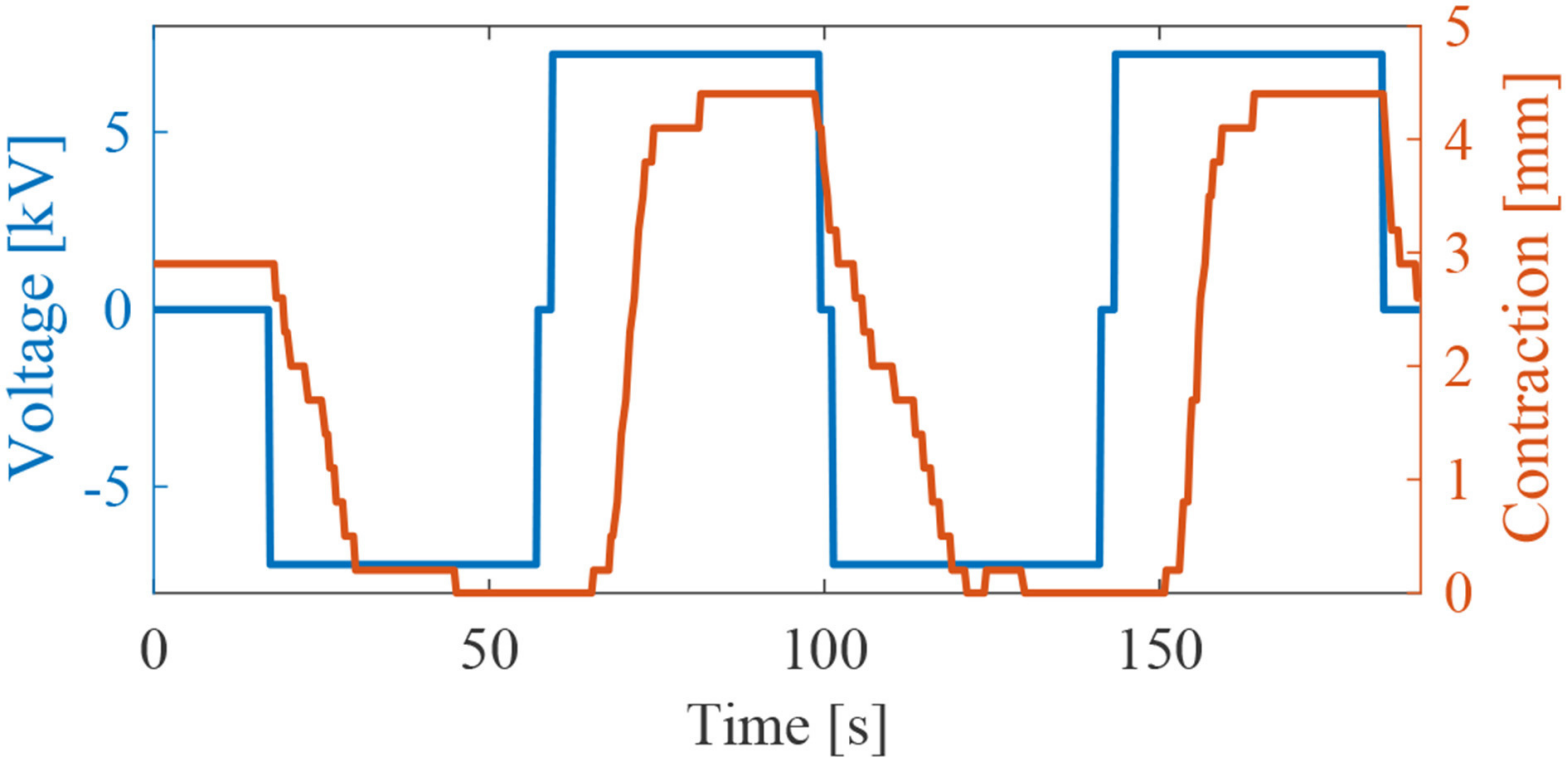
Results of the weight lifting experiment (Figure 5). Contraction of the thin McKibben muscle activated by two Stretchable Pumps at 7.2 kV.

We then measure the force vs. contraction ratio of the LPTMM driven by a Stretchable Pump (Figure 10). The Figure 11 speed of the motorized stage on the pull-tester is programmed to 0.1 mm/s. The maximum force measured is 0.84 N and the maximum contraction ratio is 2.2%, corresponding to a stroke of 4 mm. The hysteresis between the loading and unloading curve is due to both the intrinsic quasi-static characteristic of the LPTMM (Figure 8) and the dynamics of the Stretchable Pumps. In particular, in the case of unloading (actuator shortens), the volume of the muscle increases, therefore its pressure decreases until enough liquid gets pushed inside the muscle chamber by the pump. In addition to the LPTMM hysteresis, the shape of the unloading curve is then determined by: (1) the speed of contraction of the muscle, (2) the volume vs. contraction of the muscle, (3) the volume vs. pressure curve of the LPTMM (section Characterization of Volume vs. Pressure for the LPTMMs and Table 1), (4) the pressure vs. flow-rate characteristic of the pump. In the loading path (actuator elongates), the volume of the muscle decreases, so the liquid moves from the muscle to the reservoir through the pump and the tubing, while the pump keeps pushing the liquid in the opposite direction. This effect, in combination with the fluidic impedance encountered by the fluid, generates part of the overpressure observed in the loading cycle.

### Weight-Lifting Demo

We tested the ability of the Stretchable Pumps to drive the LPTMM to repeatedly lift up a small disk weighing 2 g. We connected two Stretchable Pumps in series to double their output pressure. Figure 5 shows the experimental set-up, lying in a vertical plane. When a positive voltage is applied to the pumps, they push the liquid from the reservoir to the LPTMM, which contracts and lifts the weight. When the voltage is reversed, the pumps drive the liquid from the LPTMM back into the reservoir, relaxing the actuator, and lowering the weight. We measured a total displacement Δ*x* = 4.4 mm from *V* = −7.2 kV to *V* = 7.2 kV.

## Discussion

In this work, we demonstrated the first integration of Thin McKibben Muscles and Stretchable Pumps to realize electrically-driven soft fluidic muscle fibers that are flexible and stretchable. Each of these muscles weighs 2 g and realizes a blocked force of 0.84 N and a maximum stroke of 4 mm.

Although these results are outstanding compared to other electrically-driven soft actuators, to employ these devices in wearable soft exoskeletons requires further improving their performance. The force and stroke can be both enhanced by connecting the muscles in bundles or by braiding them into active textiles (Koizumi et al., 2018; Abe et al., 2019). We expect major improvements of the performance of the Stretchable Pumps by optimizing the geometry and material of the electrode, and by tuning the electrical properties of the liquids.

The relatively high voltage required to drive the EHD soft pumps (5–8 kV) might raise concerns about safety and size of the power supply. These concerns have been addressed in a previous work on Stretchable Pumps where the authors demonstrate untethered operation using a palm sized battery-driven power supply weighting 18 g (Cacucciolo et al., 2019). Each of these portable power supplies is limited to a current output of 100 μA, widely below the human safety threshold (1–10 mA) but large enough to power up to 6 Stretchable Pumps at the same time. Nonetheless, some applications would benefit from reducing the driving voltage. Strategies to reduce the voltage include reducing the electrodes spacing and searching for combinations of electrodes and liquid materials showing higher EHD performance at lower fields.

A major limitation of these devices in their current form is the response time. We estimated a force rise time of 14 s and a fall time of 6.6 s, with 10 and 90% reference levels, in response to a voltage step of 6.8 kV (Figure 9). The response time of the system composed by pump and muscles depends from: (1) the flow-rate of the pumps; (2) the response time of the actuator; (3) the response time of the pump. From Table 1, we can estimate that 80 μl of fluid are required for a pressure of 20 kPa. Given that from earlier work (Cacucciolo et al., 2019) the maximum flow rate of the pump is *Q*_*M*_ = 100 μl/s, we can roughly estimate a time of the order of 1 s to reach the target pressure and force. The mechanical response time of the actuator is significantly shorter than 1 s, so it is not a limiting factor in this configuration. The results in Figure 9 confirm that there is no noticeable delay between the pressure applied by the pump and the force generated by the actuator. The most critical factor limiting the response time of the system is the EHD response time of the FC-40 liquid used for the Stretchable Pumps in these experiments. This liquid was chosen for its high breakdown voltage and good compatibility with PDMS, however Figure 9 shows how the time for the pressure to reach its maximum value is significantly larger than 1 s. Liquids with sub-second EHD response times have been already demonstrated for Stretchable Pumps in the authors' earlier work (Cacucciolo et al., 2019), but they have limited compatibility with PDMS. Future work will focus on finding liquid/elastomer combinations featuring long-term compatibility and fast responses.

Energy efficiency is a critical and often overlooked aspect in novel soft actuators. We estimated the efficiency of the pump η_*p*_ by dividing the average output power generated by the pump (product of average flow-rate $\bar{Q}$ and average pressure $\bar{p}$) by the total electrical power (the product of voltage *V* and average electrical current $\bar{I}$ from the power supply).

By using data from the weight lifting experiment (Figure 5 and Supplementary Video 1), we computed the average flow-rate $\bar{\;Q}=80\;\mu \text{l}/\text{s},\;$the ratio of the displaced volume during a single lifting event (1100 μl) divided by the lifting time (*t*_*l*_ = 14 s). To obtain the average pressure, we assumed for simplicity a linear variation of pressure vs. time between the start and the end of the lifting, i.e., between 0 and a maximum pressure *P_M_*($\bar{p}$ = 0.5 *p*_*M*_). We computed *p*_*M*_ from pressure characterization data of the same pumps used in the weight lifting experiments using the same voltage of 7.2 kV. *p*_*M*_ = 19 kPa is the average value over 1 s acquisition at 10 kHz, after the pressure reached a steady state value. The voltage is constant *V* = 7.2 kV and the current, $\bar{I}=17\;\mu \text{A}$ is the average of data recorded during the lifting phase of the weight lifting experiment (14 s at 10 kHz).

The resulting pump energy efficiency is $\eta_{p}=\left(\bar{Q}\;*\;\bar{p}\right)/\;\left(V\;*\;\bar{I}\right)$ = 0.75 mW/120 mW = 0.65%. We can also compute the efficiency η_*s*_ of the entire system composed of the soft pump and the soft muscle. η_*s*_ is the ratio between the gain in potential energy for the *m* = 2 g disk weight during a single lifting event (displacement Δ*x* = 4.4 mm) and the electrical energy used during a single lifting. $\eta_{s}=\left(m\;g\;\Delta x\right)/\;\left(V\;*\;\bar{I}\;*\;t_{l}\right)$ = 0.0052%. The low efficiency of the soft pump η_*p*_, in line with other novel soft actuators, is due to both losses in the EHD phenomenon itself (e.g., leakage currents, liquid circulation) and losses in the drive electronics.

Future work will aim at increasing the soft pump energy efficiency η_*p*_ through a deeper understanding of EHD, the use of new modeling tools to optimize the pump design and the selection of better materials. Future strategies to enhance the efficiency η_*s*_ include using more pump modules per soft muscle and adopting antagonistic configurations to reuse the elastic energy stored in the muscle during each cycle.

## Conclusion

This work demonstrated electrically-driven soft fluidic muscles in the form of thin fibers. The characterization of the devices shows values of force and stroke compatible with wearable applications (up to 0.8 N and 4 mm for each 2 g fiber actuator), while response time and energy efficiency require further optimization. These actuators are flexible, stretchable, modular and completely silent, opening up a path to a new class of fully integrated and comfortable wearables for human assistance and augmentation.

## Data Availability Statement

The datasets generated for this study are available on request to the corresponding author.

## Author Contributions

VC, HN, KS, and HS conceived the project. VC and HN designed, fabricated, characterized the devices, analyzed the data, and wrote the paper. KS and HS contributed to data interpretation. All authors provided feedback and agree with the final version of the manuscript.

### Conflict of Interest

VC and HS declare financial interest in form of a patent application. The remaining authors declare that the research was conducted in the absence of any commercial or financial relationships that could be construed as a potential conflict of interest.

## Supplementary Material

Supplementary Video 1 This video shows the system lifting a 2 g weight. Two Stretchable Pumps are connected in series to drive one LPTMM.
